# The patient–physician relationship: an account of the physician’s perspective

**DOI:** 10.1186/s13584-020-00375-4

**Published:** 2020-06-30

**Authors:** Ron Berger, Ben Bulmash, Netanel Drori, Ofir Ben-Assuli, Ram Herstein

**Affiliations:** 1 Max Stern Yezreel Valley College, Afula, Israel; 2 Sheffield Hallam Business School, Sheffield University Management School, Conduit Road, Sheffield, S10 3FL UK; 3 Holon Institute of Technology (HIT), Faculty of Technology Management, 52 Golomb St., 58102 Holon, Israel; 4 The College of Law and Business, Faculty of Business Administration, 26 Ben Gurion St., Ramat Gan, Israel; 5 Ono Academic College, Faculty of Business Administration, 104 Zahal Street, 55000 Kiryat Ono, Israel; 6 Department of Business Administration, The Center of Academic Studies, Or Yehuda, Israel

**Keywords:** Patient–physician relationship, Social exchange, Pragmatism, Trust, Reciprocity, Benevolence, Medical relationship, Analytical hierarchic processing

## Abstract

**Background:**

The issue of patient–physician relationships in general, and particularly the trust of patients in their primary care physician has gained much interest in academia and with practitioners in recent years. Most research on this important topic, however, focused on how patients view the relationship and not how the physicians see it. This research strives to bridge this gap, with the resolution of leading to an improved appreciation of this multifaceted relationship.

**Methods:**

A survey of 328 actively practicing physicians from all four health maintenance organizations (HMOs) in Israel resulted in a hierarchical formation of components, indicating both the relative as well as absolute importance of each component in the formation of the patient–physician relationship. The sample conducted was a convenience one. Methodologically, we used two different complementary methods of analysis, with the primary emphasis on the Analytic Hierarchical Processing (AHP), a unique and advanced statistical method.

**Results:**

The results provide a detailed picture of physicians’ attitudes toward the patient–physician relationship. Research indicates that physicians tend to consider the relationship with the patient in a rather pragmatic manner. To date, this attitude was mostly referred to intuitively, without the required rigorous investigation provided by this paper. Specifically, the results indicate that physicians tend to consider the relationship with the patient in a rather pragmatic manner. Namely, while fairness, reliability, devotion, and serviceability received high scores from physicians, social interaction, friendship, familial, as well as appreciation received the lowest scores, indicating low priority for warmth and sociability in the trust relationship from the physician’s perspective. The results showed good consistency between the AHP results and the ANOVA comparable analyses.

**Conclusions:**

In contrast to patients who traditionally stress the importance of interpersonal skills, physicians stress the significance of the technical expertise and knowledge of health providers, emphasizing the role of competence and performance. Physicians evaluate the relationship on the basis of their ability to solve problems through devotion, serviceability, reliability, and trustworthiness and disregard the “softer” interpersonal aspects such as caring, appreciation, and empathy that have been found to be important to their patients. This illustrates a mismatch in the important components of relationship building that can lead to a loss of trust, satisfaction, and repeat purchase.

**Policy implications:**

We study the impact physicians’ incentives have on the tangible relationship and discuss the significance of physician-patient relationship on satisfaction with the health service given. As a result policies leading to a more dynamic role must be given to the patient, who being well informed by the physician, can help in the decision making process. Policy schemes need to be implemented as a way of changing physicians’ behavior, forcing them to better construct and utilize this dyadic relationship.

## Introduction

Business relationships are characterized by having “service providers” and “service receivers”. In the health care service, the service providers are considered as having more power in the dyadic relationship than other types of services. This is due to the asymmetrical nature of the relationship, in which the receiver faces or perceives a high degree of complexity in the medical service being given. Trust, a key factor in the relationship between the service provider and the service receiver and is also unique in the health care service context. It is considered responsible for the placebo effect, for the effectiveness of alternative medicine, and for the inexplicable variations in the way patients respond to conventional therapies [1], furthermore there is much research undertaken today in the field of alternative / complementary medicine such as the National center for Complementary and Integrative Health.

Some researchers examined what constitutes a good physician [2–4]. It is understood that physicians’ technical knowledge and the skills are not sufficient indicators of performance in the eyes of patients [5–7]. To be a good or appreciated physician or perceived as one, it is important to have good interpersonal skills that establish strong, trust-based physician–patient interactions [8–10]. There is ample evidence that patients seek a strong relationship with their primary care physician as a result of their need for dependency and lack of knowledge [11–14].

In the past, the importance of the patient–physician relationship did not get much attention from health care decision makers [15–17]. Today, however, health care services and physicians perceive and refer to this relationship as a strategic marketing tool for three main reasons. Firstly, in the current competitive milieu, physicians’ financial success depends on their patients’ repeat business and referrals, resulting in fierce competition as is common in many other industries [5]. Secondly, patients view this service as a credence service, which is hard to evaluate objectively. As such, trust emerges as an important factor in building a strong and healthy relationship [18, 19]. Thirdly, medical research has shown that a strong relationship between the physician and the patient increases the success of the medical service given [20–22].

Traditionally, the patient–physician relationship has been structured around the concept of what can be called the ‘clinical model’, which is utilitarian and teleological in its understanding of the mutual bond. The patient is seen as having a disease produced by either an external factor or a malfunctioning structure that is the source of pain and unhappiness. The recognition and treatment of this disease, if successful, will restore the patient’s well-being. This clinical underscores much of mainstream medical practice [23]. The ‘relational model’, in contrast, focuses on the quality of the process of the patient–physician interaction. Replacing the physician’s role of being an expert providing technical expertise and knowledge to the patient who passively accepts, the relationship now becomes a partaking one for both players in the framework of which they exchange information [24]. The patient is transformed from a passive bystander into an active and integral participant in the healing process. In any joint relationship, and especially with a credence type of service, communication leading to mutual trust is a crucial element in the success of the interaction [25].

The business of medicine has shifted to concentrate on profit and technology utilization and, in parallel, to cultivating the relationship between patients and physicians to increase consumer satisfaction and maintain profits [26]. Health care costs are rising alongside a commensurate decline in patient satisfaction [27] and a growing number of complaints. Most patients’ complaints do not relate to health skills, but to ineffective communication. Most often, patients protest that physicians do not pay attention to their needs. Patients want more information about their problem and treatment outcomes, guidance on what they can do for themselves, more information on treatment side-effects, and to be an active participant in the healing process [28–30].

To gain further insights into the dyadic physician–patient relationship, the focus of this research investigates the factors influencing relationship formation from the physician’s perspective. The flow of the article is as follows. We begin by discussing the foundations of social relationship formation in the medical field and discuss its importance. These foundations are interrelated and include benevolence (affective ties), honesty (trust), and reciprocity, leading to satisfaction from the mutual relationship. This is followed by a description of the research methodology, research tools and findings. Lastly, in the Conclusion section, we discuss the differences in relationship formation between physicians and patients, and how this relationship can be better structured to facilitate better treatment resulting in mutual satisfaction. Although this research focuses only on how physicians view the relationship, using published works, we illustrate how patients see this relationship and then compare and contrast the two views to get a more holistic picture.

## Literature review

Medical diagnosis determined by the physician is a complex, bio-psycho-social one [5, 31, 32] hence, to understand the whole, the components need to be understood too. Since patients and physicians often disagree on what is perceived as “good health service” [33, 34], the goal of our study is to assess the most important aspects of the patient–physician relationship from the perspective of the physician and compare it to the views of the patients (based on existing literature). Utilizing this comparison, we can shape a more holistic view of this dyadic bond. Notably, if each party refers to different aspects of the relationship and defines it in their own way, it is essential to bridge this gap between the parties’ perceptions in order to build a stronger relationship, leading to better treatment outcome.

Identifying the constructs patients use when making physician choice decisions, and especially their evaluations of subjective quality-related choice criteria, is of increasing importance to the health care business [22]. This is because the role of the patient in the hospital and physician selection process has grown. The increase in customer choice and the inherent complexity of the medical facility choice process have become more apparent, especially with increased privatization and the profit orientation view [34]. Accordingly, to better understand and align this relationship, physicians’ views must be examined.

A variety of studies have shown that physicians and patients have different views regarding what might be effective communication between them. These views influence the perceived quality of the medical service rendered (Berger et al., [35]). Acquiring communication skills in times of change and uncertainty can lead to a competitive advantage. Medical educators should use patient–physician perceptions of care and focus on the areas of teaching that will help practitioners to meet patients’ expectations. Table 12 summarizes the differences in views of what is considered good medical service (see Appendix C).

No doubt that the asymmetries noted in the patient-physician relationship is derived mainly from the differences in health seeking behavior, and use of the internet. According to Moorhead et al. [36] several key ways that social media are being used today in healthcare: to provide information on a range of issues; to provide answers to medical questions; to facilitate dialogue between patients and health professionals; to collect data on patient experiences and opinions; to use social media as a health intervention, for health promotion and health education; to reduce illness stigma; and to provide a mechanism for online consultations. Thus, these diversified ways increase availability of health information but at the same time increase variety of opinions some objectives and some subjective.

Initially, medical journals focused on the medical act itself: interventions, hospitalization and the administrative side [37, 38]. Later, these journals started studying non-medical factors such as patient–physician relationships, organizational climate, and benefits provided by health providers outside the strict health services given [39, 40]. Over time, the patient–physician bond has evolved because of such developments as improved patient involvement in the medical procedures and doctor and/or facility choice, while reducing the passive acceptance of therapeutic indications as is. Patients’ responsibility regarding their own well-being has increased as well as the degree of information they have. Their demand for more knowledge and involvement from their physician has also grown. Numerous scholars have argued that traditional, symptomatic-oriented medicine is being replaced by patient-centered medicine where the physician invests more time on the patient’s problems and not only medical but also psychological and social ones. It was found that most patients want an active collaborative and humane involvement in the management of their own illness [16].

One study that examined the perspective of both parties was that of Krupat et al. [41], which was more qualitative in nature. The study indicated that from the perspective of patients, trust was established when power and information were shared by physicians, whereas physicians were apparently less affected by these aspects shaping patients’ attitudes. We claim that such incongruence may cause mistrust between patients and physicians, and lead to misunderstandings. The study further claimed that from the physicians’ perspective, the patient–physician relationship is rational and practical in nature. Again, this rational view by physicians is incongruent with the patients’ view, which is subjective in nature, emphasizes information sharing and empathy and takes into account their emotional state [1]. Thus, while Bendapudi et al. [2], who focused on the patient’s perspective, claimed in their qualitative research that physicians’ openness to sharing with their patients in an empathic manner all aspects of information collected leads patients to trust their physicians more, physicians may define this relationship with their patients one-sidedly. They provide their patients with information based on rational indexes and less on emotional indexes and data, an area with which they may feel less conferrable with—a point that needs to be further explored.

In many service contexts and especially in the medical industry, customers do not know the appropriate level of service required for their specific needs [42]. They rely on the advice of an “expert” who typically also provides the subsequent service. For example, the Hippocratic Oath of a physician controls for the problem of under-treatment in the medical services. The separation of physicians and pharmaceutical and other medical service providers is intended to circumvent overtreatment by unravelling the motivations to prescribe medications and market medical facilities from the revenue made by selling them. There is a continuing discussion in the health care literature about the presence of physician-induced demand [43]. Physicians may offset the drop in the number of customers by a rise in the scale and scope of care delivered in each encounter. For instance, research has shown that the incidences of cesarean deliveries compared to standard child births are linked to the remuneration differences of health insurance policies [44]. Medicine has become like any profit-oriented business, serving markets rather than patients, and focused on throughput rather than patient-centered care. This is highlighted in the CNN headline, “Patients give horror stories as cancer physician gets in 45 years” [45]. Conflicts of interest are accountable for an abundant amount of ethical wrongdoing [27]. Defensive medicine is another important issue in medical services. This term refers to physicians ordering tests and procedures, making referrals or taking other steps to help protect themselves from liability rather than to benefit their patients’ care [46]. This certainly results in the “overuse of medical services” solely to ensure that the physician is protected from a malpractice lawsuit. As a result, it is very difficult for a patient to assess if he or she is getting the right amount of medical care or is over- or under-treated. It is important to note that it is also very difficult to assess the gap, if one exists, between the level of treatment needed to the one given ad hoc or post hoc, thus increasing the perceived risks.

Value from a service rendered is frequently fashioned within the setting of the supplier–buyer relationship [47]. Relational significance is considered as the professed net worth of the tangible benefits that emerge over the period of the relationship [48]. Some goods and services, due to their complexity, can mainly be delivered within the framework of a relationship where the buyer is compelled to trust the supplier, which is characteristic of the medical service industry [49]. Medical treatments offer the most complicated and maybe the most important environment within which trust should foster. For many diseases, no satisfactory treatment exists, with others success is only random. Thus, a failing treatment is no perfect signal of under- or over-treatment. These types of goods and services are called “credence goods”—goods/services whose quality when rendered cannot be measured even after their receipt [50].

Credence goods are goods and services traded within interactions categorized by high levels of information asymmetry, where it is the supplier who regulates the buyer’s needs [51]. Many professional services have the attributes of credence goods, as they are often customized [52], requiring intensive interaction from both parties to create value [53]. Quality can neither be hypothesized nor assessed by customary approaches because of credence goods’ three characteristics: heterogeneity, intangibility and inseparability [51]. The key feature of credence goods is that consumers do not know the quality of a good or service they need or are receiving before purchasing, during its usage, or after receiving it. Perceived quality of medical treatment must, accordingly, be based on non-objective cues such as perceived trust based on the dyadic relationship created, word-of-mouth, the way a physician approaches the patient, diplomas, and how the physician’s facilities look. Medical services are often considered high risk purchases, given that the level of uncertainty and perceived risks are the highest [54]. Mitra., et al. [49] recognized that customers of credence goods endure this comparatively higher level of risk by constructing relationships and spending more time searching for information about the good providers than customers of search or experience goods. The mutual relationship model presented below elucidates this symbiosis.

The potential impact on credence goods can be seen in the results of a field experiment in the market for dental care of Gottschalk et al. [55] where an overtreatment recommendation rate of 28% and a striking heterogeneity in treatment recommendations was observed. It was found that mainly dentists with shorter waiting times are more likely to propose unnecessary treatment than others. Since patients are often left to use price as the only signal of quality, it is crucial to encourage patients to rely on recommendations from other users.

### The research model

**Fig. 1:**
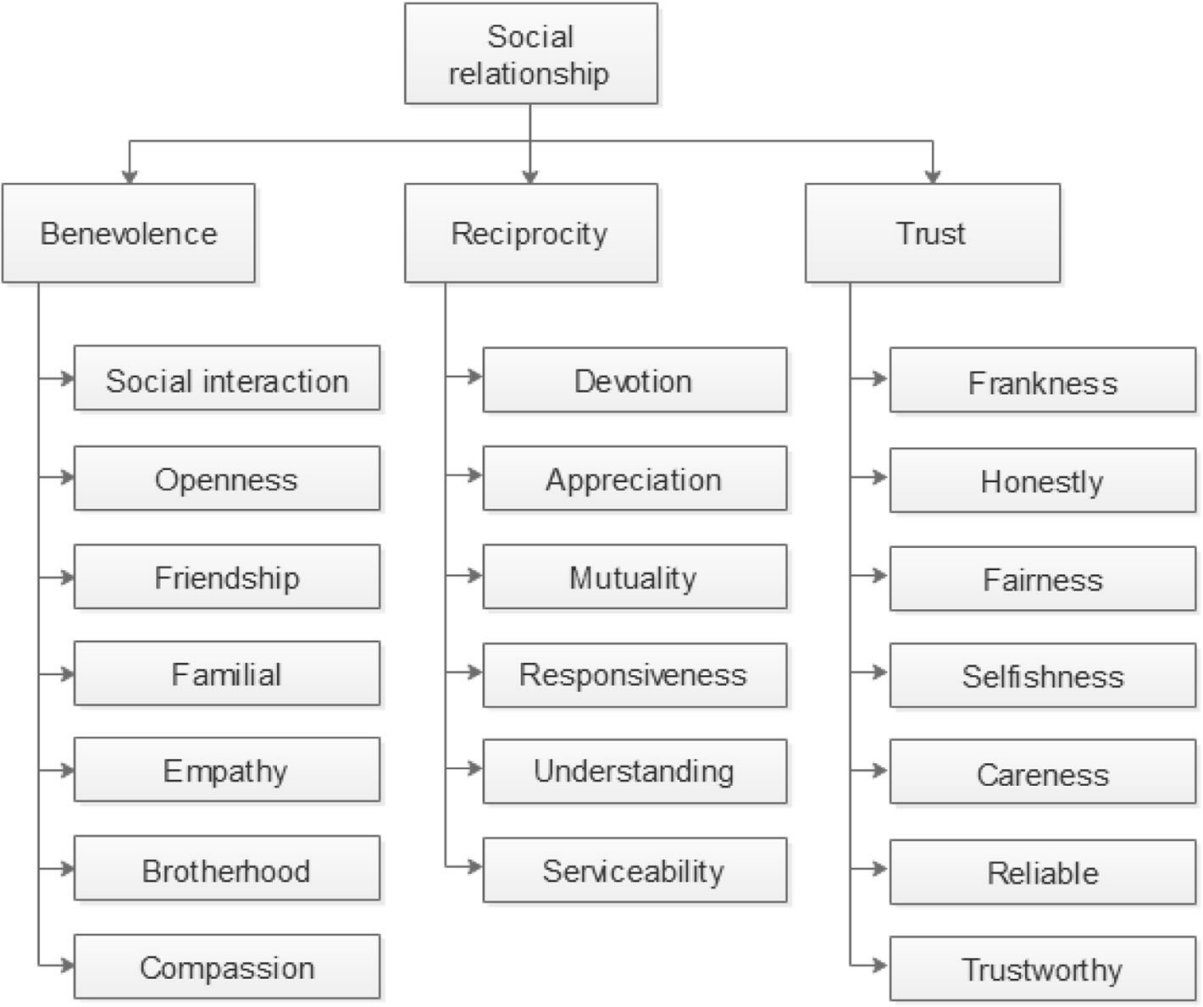
The Research Model

The relationship exploration model is commended as a research approach to circumvent service imperceptibility [56] and is suitable for exploring credence services [57]. Recent research has illustrated that social relationships are not a single-dimensional concept [58]. Instead of treating service provision as a discrete event (i.e., a one-off transaction), social exchange theory suggests treating this service as an ongoing relationship. The development of relationships is an intensive process that is costly, time-consuming, and does not necessarily generate an immediate result. To explore the individual relational constructs that together build a better picture of what is seen as a good quality relationship, we utilize the multi-dimensional GRX scale [59]. The scale consists of three main constructs: (1) benevolence (i.e., mutual feelings); (2) reciprocity; and (3) trust (i.e., trust that one will do what has been promised and that one has the ability to do what was promised). Each construct is broken down into sub constructs, as presented by Yen and Barnes [60], which facilitate better understanding of the issues, as presented in Fig. 1. The GRX scale has been used extensively to measure the quality of social relationships in many areas, for example, organizational capabilities [58], leadership [61, 62], service quality in hospitality management [63–65], business ethics [66], and customer relationships [67]. It has been further utilized in countries such as China, Russia, India, and Arab countries. A strong social relationship was found to be based upon three constructs [68]. The first construct is the confidence that the relationship partner will act benevolently to the advantage of the relationship [69]; the second is that the partner will behave honestly and can be trusted to be competent in his or her role [70]; and the third is that the partner will reciprocate the trust given, creating a long term relationship [71].

Research has shown that social relationship quality is viewed as a higher order construct comprising multiple constructs [9, 72]. When a customer feels that she is involved in a high quality relationship, she will be satisfied with the service performance and is able to rely on the service provider [42]. According to Parsons (2002), a strong relationship refers to the degree to which the performances meet customers’ expectations. Recall that in the medical profession, it is almost impossible to measure objectively the quality of service given, as it is a credence good [73]. This means that even after the service is rendered and the patient did not die or suffer adverse side-effects, it is impossible to determine that if the service had been given by another physician, the outcome could have been better. Furthermore, in many cases it takes years for side-effects to become apparent. As a result, the only way to examine satisfaction in the medical field is the patient’s perceived satisfaction, in many cases based on a personal view of the outcome. The following is the discussion of the constructs comprising our model.

#### Benevolence (affective tie)

Benevolence represents feelings and affection, a type of an emotional attachment that signals the quality of the relationship. It is claimed that in the medical industry, knowing the patient is at least as important as knowing the disease [9]. It is claimed that physicians with a warm and friendly style are more effective than physicians with a more formal style [74]. Empathy could be viewed as an aspect of medicine’s sacred commitment to stand with the sick, and the fragility of existence. It is triggered by a need for companionship and mutual understanding, especially during this exposed and unbalanced relationship. Benevolence has been found to intensify satisfaction amongst exchange partners [58]. Hence, a patient–physician relationship that demonstrates benevolence is thought to lead to better perceived care and greater patient–physician satisfaction, leading to a long-term relationship [16].

#### Trust

Trust implies credibility and, by increasing trust, one can improve relationships and increase mutual co-operation. As a rule, the patient’s trust in a physician is connected to one’s illness-generated vulnerability situation. It has been generally acknowledged that in situations in which there is a high level of perceived risk, creating trust in the service provider’s abilities necessitates a greater dependence on personal sources of information (such as friends and relatives) rather than via impersonal sources [22]. Trust in the patient–physician relationship is akin to the trust displayed in the family cell. This relationship has a robust affective and emotional dimension [43]. Trust is a necessary condition for medical practice; it is the “fundamental moral law for medicine” [75]. In the past, the trust of families and patients in a physician was imbedded. Today, trust is seen as something that is built gradually through a number of exchanges, and families and their patients are seen as active contributors in the service given based upon the capacity to manage, observe, and evaluate various circumstances concerning their health situation [76].

We define trust as prevailing when one actor has assurance in the exchange regarding the other actor’s integrity and reliability. This is when, for example, the physician can be relied upon to deliver on his promises [77]. Mutual trust in the medical profession is seen as a key relational building block. According to Hall et al. [78], trust is seen as significant in its own right because it is the construct that gives medical interactions intrinsic value. Trust can be seen as the willingness of one party to be vulnerable to a particular action that is important to the trustor, irrespective of the ability to monitor or control that other party’s actions [79]. Over the years, numerous researchers highlighted the need for trust in the health industry and stressed the importance that the patient has faith in that the physician will look out for the patient’s interests [78].

Trust is critical to patients’ willingness to seek care, reveal sensitive information, submit to treatment, and follow physicians’ instructions/recommendations [80]. Previously, the belief was that a comprehensive understanding and trust can only be built up over time through a close relationship that went beyond an understanding of the medical needs of each patient. Today, trust is viewed as a fundamental building block in the medical process. Trust is developed through repeated interactions in which the patient and one’s family observe the physician and decide if he is consistent, competent, honest, fair, responsible, and benevolent. Through engaging in actions that demonstrate honesty and extra effort, the patient develops trust in his physician [81]. Being able to measure trust is vital for physicians because it enables them to better monitor and evaluate the trust that is integral in building a strong health system with better health and economic outcomes [82]. Trust seems to work like a prognosticator of the endurance of a relationship between a certain patient and a certain physician. It can be seen as a motivating cause for increased adherence to wide-ranging medical instructions and recommendations related to the treatment, self-care actions and the inclination to pay attention to health in a sustained and continual method.

#### Reciprocity

Researchers are increasingly focusing on defining physician-to-patient communication to show that it plays a significant role in creating customer satisfaction and improving perceived quality of medical services given [22]. Dealing in uncertainty, risk, and the impossibility of generating a definition for the situation that would result in a solution for health creates the basis for the patient’s need to trust the medical system. It has been established that reciprocity is an important key construct in building a fruitful patient–physician relationship [83]. Reciprocity is when one gets compensated for one’s honesty and professionalism by, for example, repeat business and referrals [58]. This is a useful mechanism to achieve better co-operation between both parties. Even if only one member of the dyad breaks this reciprocal relationship, both parties’ interests may be damaged [59]. Reciprocity, in our context, means matching the differing viewpoints of the patient–physician communications. Given that credence services are seen to constitute high risk purchases [57], this would logically imply that the reciprocity construct would take on greater significance in the evaluation of medical services. Most health service providers identify the significance of building more sustainable and long-lasting relationships with their patients [77]. Very often, increased customer loyalty and repeat purchases are argued to be the single most important driver of a firm’s long-term financial performance and the medical industry is no different. We believe that if the physician would go that “extra mile” for the patient, the patient would reciprocate and help build a long-lasting relationship by coming back to the same physician for further treatment, hence, increasing performance and mutual satisfaction.

## Methods

In this study we adapted a new approach in measuring the constructs of the patient–physician relationship in the medical field. The constructs created are based on the well-known GRX model that focused on B2B marketing relationships [58, 59]. The basis of the model stipulates that, first, the two parties (i.e., physicians and patients) wish to develop a relationship. Thus, items that indicate long-term interest in the mutual relationship must be measured. Second, as it is a credence good, the quality of the mutual relationship can mainly be measured subjectively. In our study, the data was analyzed by utilizing complementary research methods. The first relies on Fisher’s ANOVA for related samples, referred to as the semantic scale method, with post hoc t-test analyses. The second method is the AHP (analytical hierarchy processing) method developed by Saaty [84]. Below we focus on explaining the AHP method since the ANOVA method is well-known in the social sciences field.

### The semantic scale and the AHP method

The semantic scale method is a simple and commonly used method. In this study, a list of relationship-related components was presented to physicians, and they were asked to rank each component on a 1–5 scale (5 indicating very high impact and 1 indicating very low impact on the relationship). This method can also be referred to as ‘The Absolute’ method, since each component receives an absolute value; this is in contrast to the AHP method. The ranking results were analyzed using Fisher’s ANOVA for related samples.

The AHP method was originally developed to solve problems with many variables. This method consists of a structured ranking system, which reflects hierarchically the relative value of a set of components, regardless of whether these components are goals, objectives, people’s desires, or, as in our case, indicators of an individual’s social relationship experience. AHP requires executing the following steps: (1) Identifying the factors or components that are appropriate for the problem at hand. These factors can be organized hierarchically as well, hence, this method allows the researcher to arrange the components in groups in a logical fashion. (2) Comparing pairs of information components, and deriving a quantified value for each comparison. Grade 1 denotes equality between the two components compared. Grade 9 to either direction denotes absolute preference for one of the two possibilities. (3) Making an adjusted calculation, which pertains to the mathematical calculation of all the comparisons. (4) Interpreting the ranking reached. It should be noted that this pairwise comparison procedure invites inconsistencies, which can be measured using an inconsistency coefficient. This method can be referred to as ‘The Relative’ method, since each component is evaluated relatively to all other components.

This advanced method provides the researcher with a way to take into account the complexity of a large number of component relationships. Namely, while ‘The Absolute’ method treats each information component separately, in a rather isolated fashion, ‘The Relative’ method promotes a bigger-picture approach, which enables the researcher to evaluate the components within a broader context. Additionally, it should be noted that the AHP method is directly aligned with the question of which information component is more or most important; this is where our study focus lies. In opposition, ‘The Absolute’ method asks the question: how important is each information component? Thus, it only indirectly indicates the relative importance of information components. The AHP method models the whole social relationship concept (shown in Fig. 1 below) accurately. It considers all relationships between the components and the existing hierarchy of the research model.

This unique method has been used in prior work in health evaluation, assessment of investment alternatives, and modeling and assessing financial decision making [85, 86] and also in modeling within the health care arena [72]. Figure 1 below presents the components of interest in our model in a hierarchical form, with the social relationship quality at the top, followed by the three sub-categories (constructs) of benevolence, reciprocity, and trust.

### Data collection

Our research into the patient–physician relationship uses data drawn from health care facilities in Israel. Medical tourists come to Israel for treatments that cost significantly less than in many other developed nations with the same quality level. Some seek relief for a variety of medical conditions at hospitals, treatment centers, and spas such as those located at the Dead Sea, a world-famous therapeutic resort [87, 88]. In a survey of 25 countries, the Medical Tourism Index (MTI) positioned Israel at the top, as the most prominent destination for medical tourism for services, care, and the finest patient experiences, and third overall as the best place for non-Israelis to get medical care. Its hospitals are continuously ranked among the best in the world [89]. As such, we see it as a good base to conduct our research.

We conducted a study of active physicians from four different HMOs in Israel. The physicians were approached at various medical seminars conducted in Israel. The physicians filled out the questionnaires during semi-structured interviews (to validate and clarify the responses and increase the response rates) and took an average of 30 min to compete the questionnaire. In addition to the background questions (age, tenure, gender), the survey included 23 items ranging ‘completely disagree’ to ‘strongly agree’ and a set of 60 item-comparisons between the information components, in accordance with model presented in Fig. 1.

About 90% of the physicians who were asked to participate agreed to be interviewed. 328 physicians responded to all items of the questionnaire (no missing data). However, 37 responses were considered suspiciously inappropriate due to survey straightlining, where participants repeated the very same answer throughout the questionnaire, while disregarding the specific questions in hand. Such response pattern implies respondents’ impatience or lack of interest. All final 291 responses were considered valid and did not have any missing data.

The characteristics of the sample show high level of representativeness of the total Israeli physician population in terms of gender, age, group of population and place of getting their degree. In terms of gender, 55% were male and 45% were female (the total Israeli physician population regarding gender is 59% male and 49% female). In terms of age, 25% were below 39, 70% were 40 to 64, and 5% were above 65 (the total Israeli physician population regarding age is 22% below 39, 73% - between 40 and 64, and 5% - above 65). In terms of group of population, 92% were Jews and 8% were Arabs (the total Israeli physician population regarding group of population is 90% Jews and 10% Arabs). In terms of place of getting their degree, 33% were from Israel, 40% were from the former Soviet Union, 20% were from West-Europe and 7% were from North America (the total Israeli physician population regarding place of getting their degree is 36% from Israel, 35% from former Soviet Union, 24% West-Europe, and 5% from North America).

## Results

**Fig. 2:**
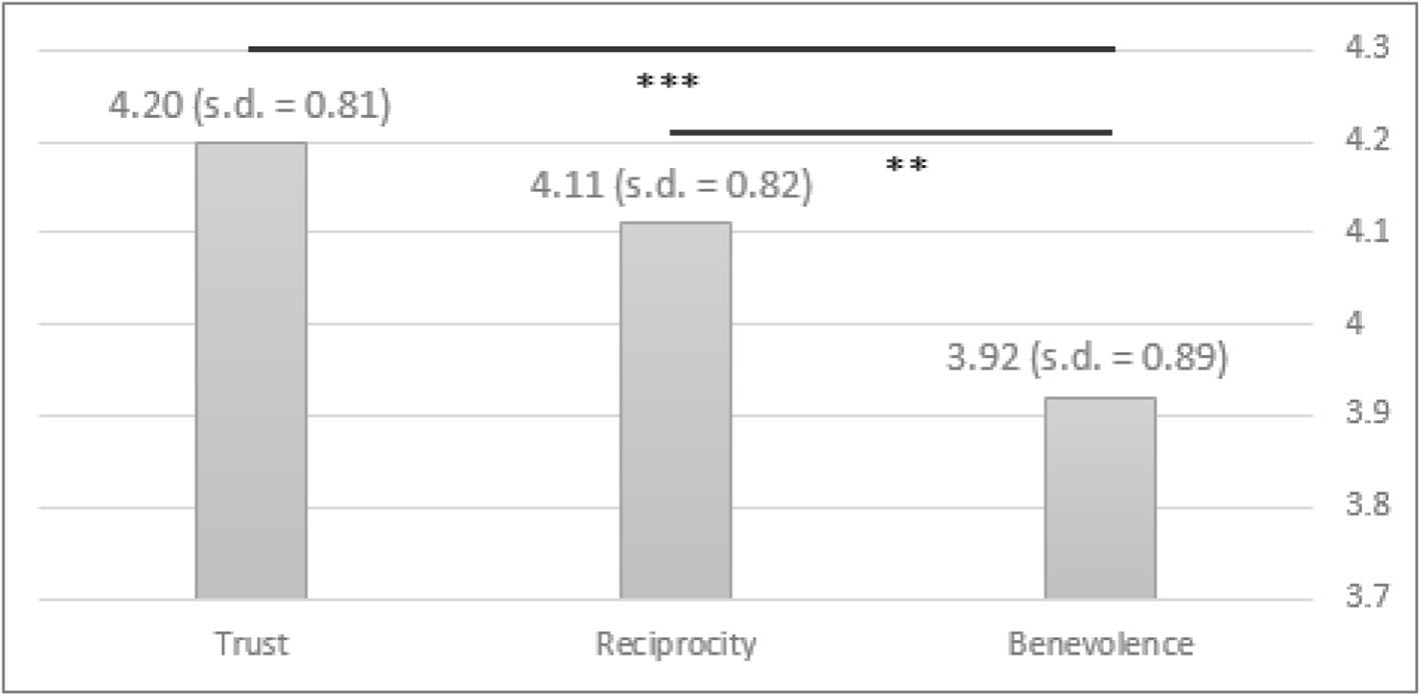
Results of Level 1 component assessment, using the semantic scale method

As presented earlier, the study model has two hierarchical levels. First, we analyzed Level 1 (see Fig. 1), which pertains to the importance of high-order components of Benevolence, Reciprocity, and Trust, in the formation of the patient–physician trusting relationship. The analysis shows that according to the two methods of analysis, Trust was considered the most important factor, followed by Reciprocity and Benevolence. Regarding the semantic scale method (see Fig. 2), Fisher’s ANOVA for related samples indicates a significant difference between the components (χ2(2) = 16.141, *p* < 0.001). Post hoc t-test analyses with a Bonferroni correction were applied and indicated that there was no significant difference between Trust and Reciprocity (*t* = − 1.289, *p* = 0.198). There was a statistically significant difference between Benevolence and Trust (*t* = − 4.233, *p* < 0.001) as well as Reciprocity (*t* = − 3.162, *p* = 0.002). Supporting these findings, the AHP analysis produced the same ranking, wherein Trust and Reciprocity showed the highest importance (with a relative weight of 0.503 and 0.383 respectively), with Benevolence having a lower relative weight (0.114). The consistency index was 0.109 in the Level 1 AHP analysis, suggesting reasonably consist comparisons among all sub-components. Notably, the task of multiple comparisons is quite demanding of attention and effort, hence, as explained earlier, a few unreasonable sets of responses (i.e. straightlining) were entirely excluded from our sample, thus leading to reasonable consistency indices throughout the study.

In the supplementary analysis, when the sample of physicians was split according to gender, female physicians ranked Trust higher than the other two Level 1 components, while male physicians valued Trust and Reciprocity equally (see Tables 4 and 5 in the Appendix B). When seniority differences were tested (not shown in the paper), no significant differences were found in all our analyses.

**Fig. 3:**
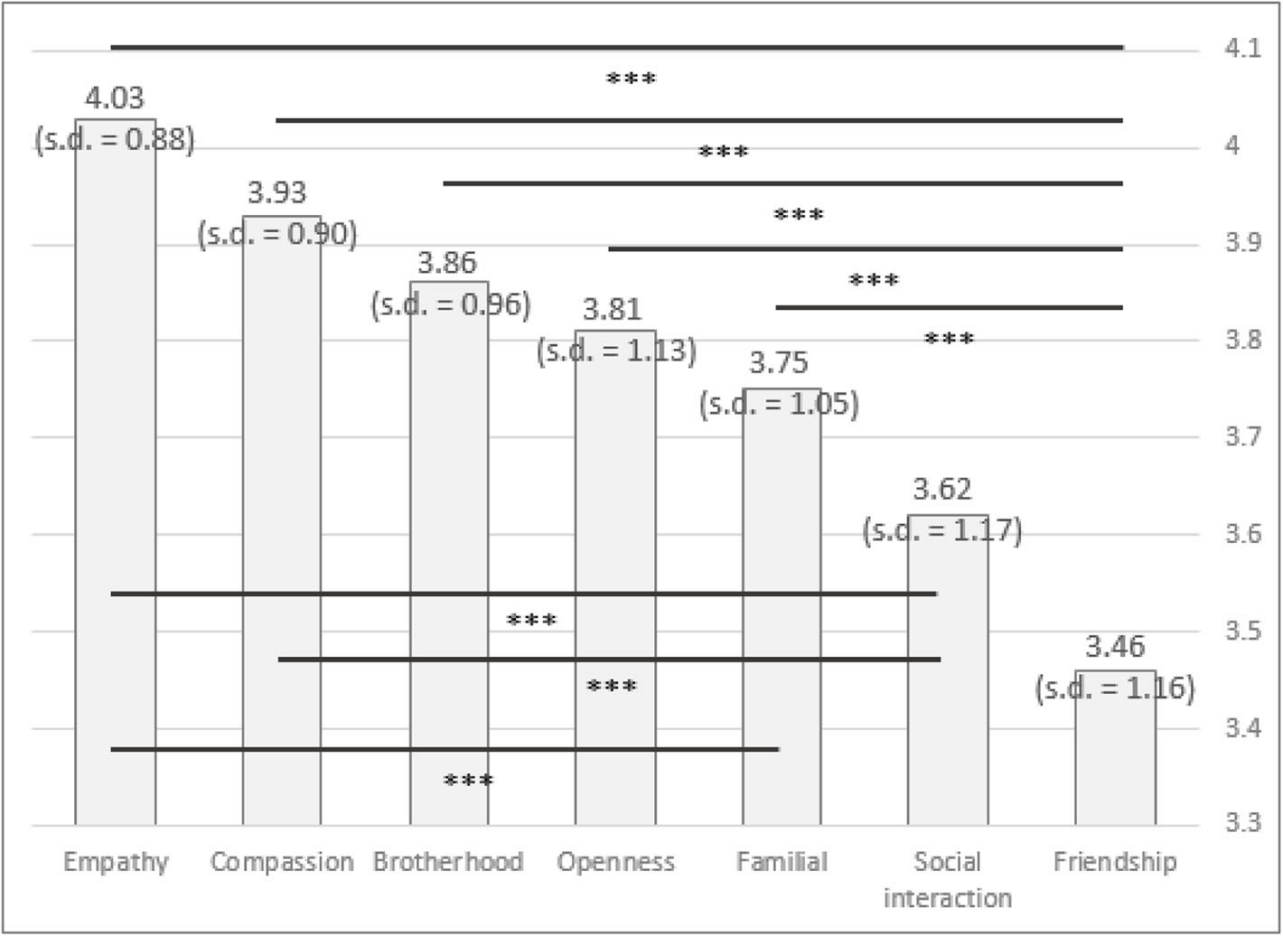
Results of the sub-components for Benevolence, using the semantic scale method

Next, each of the three Level 1 components were tested separately for their sub-components. The first component tested was Benevolence with seven sub-components. Figure 3 shows the sub-components ranked by their importance, along with means, standard deviations, and ANOVA’s post-hoc analyses findings. The most important sub-components for Benevolence were Empathy and Compassion, with Social Interactions and Friendship considered the least important. Fisher’s ANOVA identified significant differences in the means (χ2(2) = 56.959, *p* < 0.001). Using a Bonferroni adjustment, with a total of 21 comparisons, a significant difference was evident at *p* < 0.0024 (rather than at *p* < 0.05). Several significance differences were found. First, Friendship had a significantly lower value than all components except for Social Interactions (t ranges − 3.821 to − 7.224, *p* < 0.001). Social Interactions had significantly lower importance than both Empathy and Compassion (t ranges from − 4.936 to − 3.966, *p* < 0.001). The Familial component showed significant difference from Empathy (t = − 3.953, *p* < 0.001), as shown in Fig. 3.

By and large, the AHP method (Table 1 in Appendix A) confirmed the results of the semantic scale method, with the highest scores for Compassion, Brotherhood, and Empathy, with a low value for social interaction. One exception that can be seen—Friendship—which received a relatively higher rating. Nonetheless, the overall results are similar in both methods of analysis. There were no major differences when the data was split by gender (see Tables 6 and 7 in Appendix B).

**Fig. 4:**
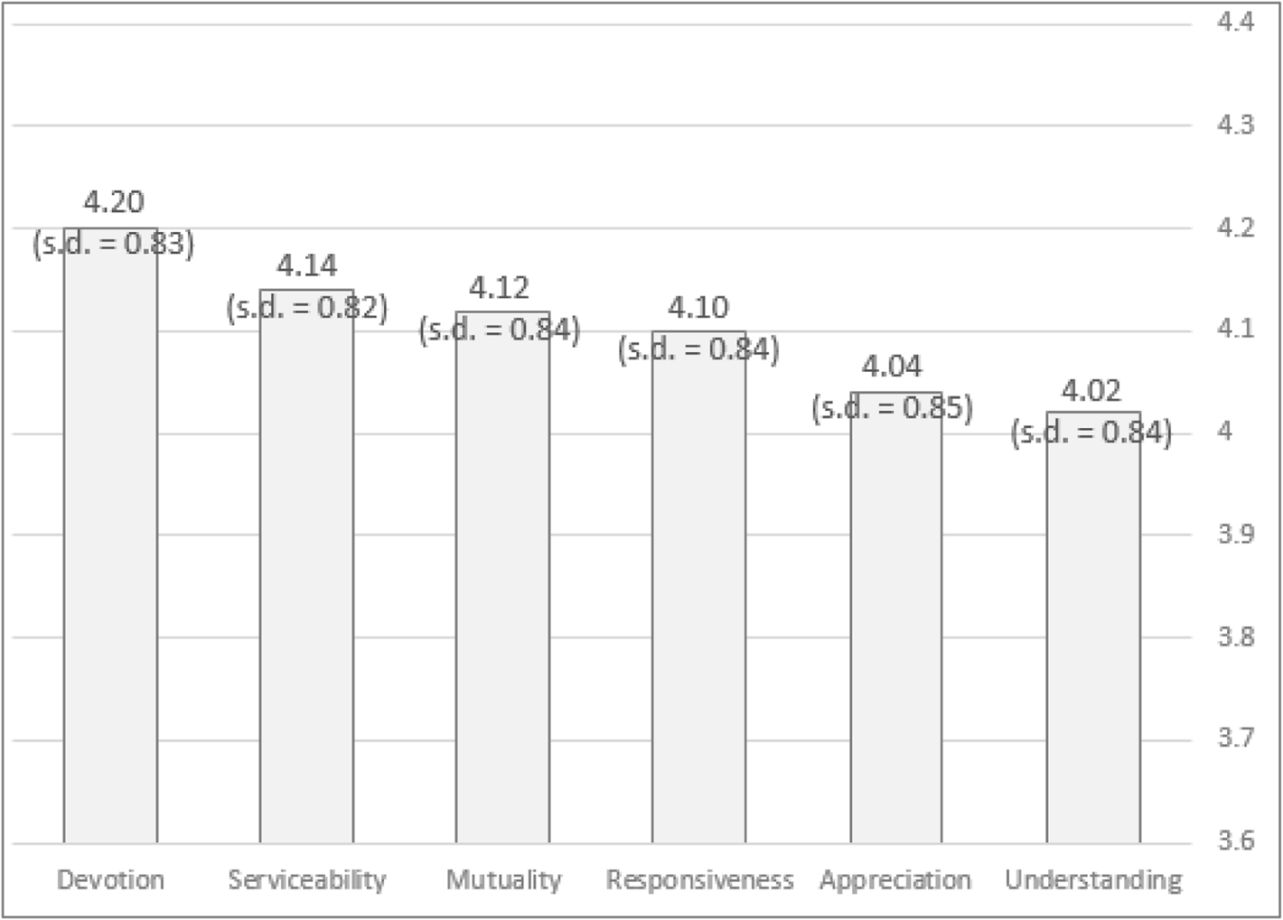
Results of the sub-components for Reciprocity, using the semantic scale method

The second component tested was Reciprocity with six sub-components. Figure 4 below shows the sub-components ranked by relative importance. Devotion received the highest score, while Appreciation and Understanding received the lowest scores. Notably, the differences in importance between the sub-components of Reciprocity (ranging from 4.02 to 4.20) are smaller than the range of values found for Benevolence (ranging from 3.46 to 4.03). Thus, while according to the ANOVA calculation, there is a statistically significant difference (χ2(5) = 12.354, *p* = 0.030), with a total of 15 comparisons. A significant difference was seen only at *p* < 0.0033; hence, no statistically significance can be said to have been found.

The AHP results presented in Table 2 in Appendix A clearly support the results found in the semantic scale method, with Devotion and Serviceability having the highest relative weight (0.235 and 0.264 respectively) and Understanding and Appreciation having the lowest weight (0.050 and 0.075 respectively). Notably, the differences between the sub-components are more evident in the AHP analysis.

When the data was split by gender, there were certain significant differences in regard to some components such as Serviceability, which was given the highest importance by females, but a lower one by male physicians (F (6, 284) = 2.452, *p* = .025; Wilk’s Λ = 0.951). The results split by gender are presented in the supplementary analysis in Appendix B (Tables 8-9).

**Fig. 5:**
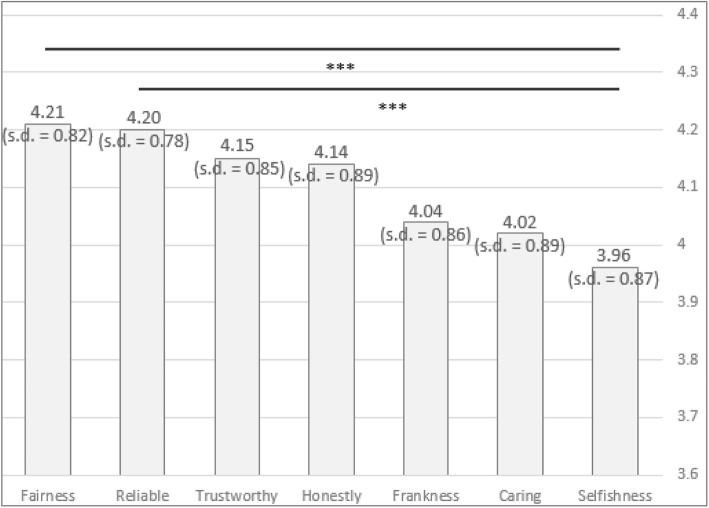
Results of the sub-components for Reciprocity, using the semantic scale method

Lastly, the third component, Trust, which was ranked the highest among the three components, has seven sub-components. Figure 5 presents the relative importance of each sub-component. Similar to Reciprocity, the range of values (3.96 to 4.21) is not large in comparison to Benevolence. Fischer ANOVA test for related samples indicates a difference in the means (χ2(6) = 31.640, *p* < 0.001). Here a significant difference was considered at *p* < 0.0024 due to multiple t-test comparisons. The results show that Fairness (t = − 3.785, *p* < 0.001) and Reliable (t = − 3.545, *p* < 0.001) are significantly more important components than Selfishness, the least important sub-component.

In contrast to all prior results, here the AHP results (Table 3 in Appendix A) did not exactly match the ranking provided by the semantic scale method. According to Table 3 in Appendix A, Honestly and Fairness were considered the least important, while Trustworthy, Reliable and Caring were considered the most important. It is also notable that the consistency index for Trust is higher compared to Benevolence and Reciprocity, suggesting lower consistency in sub-component comparisons. Therefore, it may be the case that for the Trust component, the physicians had difficulty differentiating between the underlying meanings of the sub-components. Indeed, it may be quite challenging to clearly distinguish between Trustworthy, Honestly, and Frankness, for instance. We view this as a possible explanation for this inconsistency.

Finally, gender did not play a significant role in the sub-component rankings (see Tables 10 and 11 in Appendix B).

## Discussion

Health care is an issue dominating the landscape of public discourse and debate today. Rising health care costs and insurance premiums make accessibility to good health care a hefty challenge for many patients and even put it out of reach for some unfortunate people who have to rely on a deteriorating public health system [90]. In the twenty-first century, medicine is evolving rapidly, focusing on technology and profit. As a result, nowadays health care systems, hospitals and insurers have moved the focus to patient satisfaction [91]. It is generally determined by patient satisfaction surveys and does not consider physicians’ or other medical staff’s perceptions of the service given. Research has shown that stressing improved patient satisfaction, much like defensive medicine, actually leads to more unnecessary tests being performed, harmful drugs being prescribed, increased hospital visits, increased health care cost, and increased morbidity and mortality [90].

Given these trends, patient involvement in the care process is ever more important. Many physicians are calling for shared decision making, so that patients take part in their own health care and understand better the risks associated with various procedures and drugs. This is important as there are risks associated with almost all treatments and medications. With the focus on customer satisfaction today, it makes little sense in today’s medical marketing philosophies for physicians to make medical decisions without sufficient input from patients—presenting additional confirmation of the need for a strong dyadic relationship.

Essentially, efficient patient–physician communications—a major patient–physician relationship component [92]—must become a priority. Within their increasingly dyadic relationship, many patient complaints do not relate to the physician’s skill set, but to ineffective communication leading to misunderstandings and decreased satisfaction from the service rendered. Most often, patients complain that physicians do not listen to them [87]. Patients want more information about their problem and treatment outcomes, more information on the side-effects of the treatment, and advice on what they can do for themselves.

## Conclusions

The main objective of this study was to explore and clarify the way in which physicians perceive the mutual relationship they share with their patients. Specifically, the aim was to quantify the perceived impact of an array of information components based on the GRX scale [58]. In this study, we used two different methods of analysis, which together provide highly consistent and, therefore, robust results regarding physicians’ perspective on the patient–physician relationship. Interestingly, our findings show that in contrast to patients who traditionally stress the importance of interpersonal skills [5, 9, 74], physicians in our sample stress the significance of the technical expertise and knowledge of health providers, emphasizing the role of competence and performance in the trust relationship. This illustrates a mismatch in the important components of relationship building that can lead to a loss of trust, satisfaction, and repeat purchase.

Our results show that physicians rank benevolence as less important than trust and reciprocity, with social interaction and friendship sub-components as the least important components in the relationship. Rather, physicians evaluate the relationship on the basis of the physician’s ability to solve the patient’s problems through devotion, serviceability, reliability, and trustworthiness. This bottom-line problem-focused approach [93] toward the relationship seems to indicate that physicians disregard, to an extent, “softer” interpersonal aspects such as caring, appreciation, and empathy. Interestingly, these findings were consistent overall across genders and tenure, creating a clearer picture of the physician’s viewpoint of the relationship. The use of two different means of analysis strengthens the validity of our results. The results highlight, therefore, the gap in how patients and physicians experience and evaluate trust in their care relationship. While our research shows that patients stress the importance of their feelings and emotions, physicians pragmatically believe their job performance is the most significant indicator for trust in the relationship. Interestingly, none of the physicians interviewed considered “know-how” in terms of their understanding of their patient’s feelings a source of value.

We make a unique contribution to management and primary care policy and practice, offering a new model and revised scales from the management, psychology, and health care literature. Our findings also provide a broader framework for examining and understanding the breadth and meaning of the patient–physician relationship and care management from the physicians’ viewpoint. We use a two-tier analysis to understand the constructs and sub-constructs that create this relationship better.

Previous studies have shown that improving physician-to-patient communication has an important role in increasing customer satisfaction and increasing the quality of medical services rendered [94]. The primary objective of this study was to identify the dimensions of value in professional medical service relationships focusing on patient–physician relationships and to identify the physician constructs in building a relationship with patients. Our research showed that trust made the largest contribution to patient–physician perceived satisfaction and that the relationship between trust and physician performance was strong. Physicians’ interpersonal abilities and skills were also regarded by physicians to be important considerations in determining physician performance. Patient satisfaction studies show that along with physicians’ medical skills, qualities such as listening and interpersonal skills are highly rated. Empathy, communication ease, friendship, trust, and commitment are all highly valued by patients. ‘Problematic’ physicians were more likely to be perceived as being unavailable and incapable of handling their patient’s medical complaints [87], hence, the need for physicians to manage and synchronize their communication skills.

The doctor-patient relationship involves vulnerability and trust as a result it has become one of the most important factors for patient satisfaction. However, this relationship and the encounters that flow from it are not always perfect. The quality of communication between doctor and patient involves assessment of the doctor’s willingness to include a patient in the decision-making process and to provide a patient with information. As there is a growing attention and changing policy with respect to informed consent, the quality of doctor-patient communication must be better understood. To ultimately improve longer-term patient outcomes, mutual communicative barriers must be overcome. Having presented the dyadic relationship between physicians and patients we highlight a number of possible policy implications. First, we consider the impact physicians’ incentives have on the actual relationship. Second, we discuss the importance of physician-patient relationship on satisfaction with the health service given. Trust and reciprocity in the physician are the two most important factors in explaining the variation in overall patient satisfaction more than any other aspects in the eyes of the physician. As a result a more active role must be given to the patient, who being well informed by the doctor, can help in the decision making process. Policy schemes need to be implemented as a way of changing physicians’ behavior, forcing them to better build and utilize this dyadic relationship.

There are certain limitations to the research that should be noted. First, the framework used pertains only to the perceived value of relationship components. The objective value (actual performance) of these components was not examined. Second, both methods of analysis are not error-free. The AHP method, for instance, allows for a certain amount of inconsistency, especially in cases where information components are close in their underlying meanings. To deal with this issue, we used two different methods of analysis, which showed overall consistent results. More so, the consistency levels for the AHP were reported for each analysis conducted. Third, physicians’ background was not accounted for in our analyses. While the role of gender and tenure were accounted for in the supplementary analysis, other, possibly interacting, factors could be included such as age, race, religiousness, and specialty. Also, macro-level factors such as geographic region, socio-cultural characteristics, and differences between health organization policies could be further examined. Likewise, the physicians’ mental state could also be considered in future work and measured using the GRX scale. It is possible that physicians who experience high stress levels or burnout will engage in the patient–physician relationship in a different, more distant, fashion. Fourth, there could also be a language barrier between what is understood by the physicians and what the researchers where looking to express. This mas mitigated as much as possible when building the questionnaire and with a researcher available during the research itself. Convenience sampling has advantages in terms of comfort and speed. However, the method has also shortcomings as it enables collecting data from subjects who are more available and accessible [95]. Lastly, this research was conducted in a single location that may limit its generalizability.

## Data Availability

The datasets generated and analyzed during the current study are not publicly available but are available from the corresponding author on reasonable request.

## Appendix A

### AHP analyses results

**Table 1:** Results of the sub-components for Benevolence, using the AHP relative assessment method

Information component	Relative weight
Empathy	0.159
Compassion	0.240
Brotherhood	0.219
Openness	0.073
Familial	0.138
Social Interaction	0.037
Friendship	0.135

Consistency index = 0.193

**Table 2:** Results of the sub-components for Reciprocity, using the AHP relative assessment method

Information component	Relative weight
Devotion	0.235
Serviceability	0.264
Mutuality	0.156
Responsiveness	0.220
Appreciation	0.050
Understanding	0.075

Consistency index = 0.144

**Table 3:** Results of the sub-components for Trust, using the AHP relative assessment method

Information component	Relative weight
Fairness	0.074
Reliable	0.200
Trustworthy	0.232
Honestly	0.040
Frankness	0.136
Caring	0.176
Selfishness	0.142

Consistency index = 0.204

## Appendix B

### Supplementary Analyses

#### Breakdown of the study results by gender

**Table 4:** Results of the component assessment with a breakdown by gender, semantic scale

Information component	Females (*N* = 184)	Males (*N* = 107)
Trust	4.18	4.22
Reciprocity	4.04	4.25
Benevolence	3.86	4.02

**Table 5:** Results of the AHP relative assessment with a breakdown by gender

Information component	Relative weight	
	Females (*N* = 15)	Males (*N* = 5)
Trust	0.459	0.444
Reciprocity	0.408	0.444
Benevolence	0.134	0.111

Consistency index for males < 0.100, Consistency index for females = 0.184

**Table 6:** Results of the sub-components for Benevolence with a breakdown by gender, semantic scale

Information component	Females (*N* = 184)	Males (*N* = 107)
Empathy	4.04	4.00
Compassion	3.95	3.90
Brotherhood	3.89	3.79
Openness	3.78	3.86
Familial	3.73	3.77
Social interactions	3.62	3.64
Friendship	3.48	3.44

**Table 7:** Results of the AHP relative comparison of sub-components for Benevolence with a breakdown by gender

Information component	Relative weight	
	Females (N = 15)	Males (*N* = 5)
Empathy	0.153	0.172
Compassion	0.235	0.204
Brotherhood	0.213	0.229
Openness	0.077	0.074
Familial	0.143	0.125
Social interactions	0.039	0.049
Friendship	0.139	0.147

Consistency index for males = 0.280, Consistency index for females = 0.179

**Table 8:** Results of the sub-components for Reciprocity with a breakdown by gender, semantic scale

Information component	Females (*N* = 184)	Males (*N* = 107)
Devotion	4.13	4.33
Serviceability	4.17	4.07
Mutuality	4.04	4.26
Responsiveness	4.00	4.28
Appreciation	4.02	4.08
Understanding	4.01	4.04

**Table 9:** Results of the AHP relative comparison of sub-components for Reciprocity with a breakdown by gender

Information component	Relative weight	
	Females (N = 15)	Males (*N* = 5)
Devotion	0.237	0.261
Serviceability	0.266	0.285
Mutuality	0.158	0.121
Responsiveness	0.213	0.227
Appreciation	0.050	0.042
Understanding	0.075	0.065

Consistency index for males = 0.130, Consistency index for females = 0.137

**Table 10:** Results of the sub-components for Trust with a breakdown by gender, semantic scale

Information component	Females (*N* = 184)	Males (*N* = 107)
Fairness	4.22	4.19
Reliable	4.24	4.14
Honestly	4.13	4.17
Trustworthy	4.14	4.16
Caring	3.98	4.08
Frankness	4.02	4.08
Selfishness	3.91	4.04

**Table 11:** Results of the AHP relative comparison of sub-components for Trust with a breakdown by gender

Information component	Relative weight	
	Females (N = 15)	Males (N = 5)
Fairness	0.074	0.073
Reliable	0.221	0.158
Honestly	0.042	0.046
Trustworthy	0.221	0.245
Caring	0.176	0.188
Frankness	0.123	0.176
Selfishness	0.144	0.114

Consistency index for males = 0.292, Consistency index for females = 0.196

## Appendix C

**Table 12:** Summary of the Different Views of the Dyadic Relationship

Construct	Patient–Physician Patient’s Perspective	Source	Patient–Physician Physician’s Perspective
Model	Relationship	[23, 37, 40]	Clinical
Care	Holistic: Patient centered	[2, 37]	Focused: Illness centered
Trust	Emotionally based – Built when information is shared	[1, 41]	Rationally based – Founded on one-sided Communication (the doctor dispenses advice)
Communication	Emotional – Based on empathy	[10]	Rational – Based on facts
Relationship	Reactive and dynamic	[20]	Passive – Based on medical needs
Responsibility	Mutual	[16]	Physician
Skill	Interpersonal – Emotionally based	[5, 9, 74]	Technical – Competence based
